# Diagnosis and Management of Merkel Cell Carcinoma of the Head and Neck: Current Trends and Controversies

**DOI:** 10.3390/cancers6031256

**Published:** 2014-06-27

**Authors:** Mark S. Swanson, Uttam K. Sinha

**Affiliations:** Department of Otolaryngology—Head and Neck Surgery, University of Southern California, 1520 San Pablo St, Suite 4600, Los Angeles, CA 90033, USA; E-Mail: mark.swanson@med.usc.edu

**Keywords:** merkel cell carcinoma, skin malignancy, cutaneous neoplasm, head and neck cancer, cutaneous malignancy, skin cancer, neuroendocrine tumor

## Abstract

Merkel cell carcinoma is an aggressive neuroendocrine cutaneous malignancy with a predilection for regional and distant metastasis. This malignancy presents most commonly on the head and neck of elderly Caucasian males, with a higher prevalence in the immunosuppressed. A high index of suspicion must be maintained due to the often asymptomatic presentation. Lip tumors, scalp tumors, local invasion, nodal metastasis, distant metastasis, and lymphovascular invasion are poor prognostic factors. Up to 8.7% of patients present with distant metastasis, and PET-CT is an accurate staging tool with a 90% sensitivity. Combined aggressive surgical resection with adjuvant radiotherapy affords the best regional control rates. The regional lymphatics must be addressed with either sentinel lymph node biopsy, surgery, or elective radiation due to the risk of occult metastasis. Addition of chemotherapy has no proven benefit in locoregional control.

## 1. Introduction

Merkel cells are afferent sensory receptors found in the basal layer of the epidermis that function as mechanoreceptors [1]. It is debated whether Merkel cell carcinoma (MCC) arises from malignant transformation of a Merkel cell [2] or from a pluripotent stem cell [3]. It is a neuroendocrine tumor found most frequently in the head and neck of fair-skin elderly males [4]. Like other cutaneous malignancies, it is found frequently in sun exposed areas with an increased incidence in the immunosuppressed patient [5,6]. Although uncommon, it is growing in clinical significance due to the 4-fold increased incidence from 1986 to 2006 [7]. This skin malignancy has a high propensity for lymph node metastasis, distant metastasis, and recurrence, which portends a poor prognosis. MCC is frequently compared to melanoma due its poor prognosis and aggressive behavior, as such multimodality therapy is the norm. Due to the rarity of MCC, there is debate about best practices, and this review addresses the current literature involving epidemiology, diagnostics, treatment, and outcomes with a focus on head and neck sites.

## 2. Epidemiology

MCC of all body sites has an incidence of 0.6 per 100,000, which has been increasing over the past 20 years [7]. A polyomavirus known as Merkel Cell Polyomavirus is often isolated in tumor specimens, with an 80% prevalence in Merkel cell carcinoma [6,8,9,10,11,12]. This virus has been implicated as both a causative agent and as a highly prevalent virus found incidentally, however this virus is detected in only 16% of healthy skin samples [13,14]. Environmental factors such as UV-B exposure, immunosuppression, and viral mutations may promote tumorigenesis. Although many tumors harbor Merkel Cell Polyomavirus, there is conflicting data about the prognostic implications of seropositivity on treatment outcomes.

The SEER database, which represents 26% of the population of the United States including 4,376 patients with MCC, demonstrated that MCC presents in the head and neck most commonly (41.8% of patients) followed by the upper extremities (24.6%) [4,15]. Of these lesions 81% were located in sun-exposed regions, 59% of patients were male, 90%–95% were Caucasian, and 90% were older than 50 years [4,16]. Head and neck tumors tended to present as smaller lesions but with a higher propensity for regional lymph node metastasis (50.1%) and invasion into bone, cartilage, or muscle (7.9%). Of these lesions, lip tumors had the highest propensity for local invasion (89.8%), scalp tumors presented with larger tumors with the highest risk for distant metastasis (8.7%), and external ear canal tumors had the highest rate of nodal metastasis (63.2%) [4].

The acronym “AEIOU” can be applied to describe the clinical features of MCC: asymptomatic/non-tender, expanding rapidly, immune suppressed, older than 50, and ultraviolet exposed fair skin [13,16]. Due to the often innocuous appearance, 56% of these lesions were thought to be benign prior to biopsy [16]. Immunocompromised patients including those with organ transplantation, acquired immune deficiency syndrome, or autoimmune disease have a 13.4% relative risk compared to the general population [17]. These lesions are typically red or blue with telangiectasias, often with the epidermis being uninvolved. Large tumors may ulcerate, however this is uncommon [13]. Due to often innocuous features and asymptomatic presentation, a high index of suspicion must be maintained, especially in the setting of enlarged regional lymph nodes.

### 2.1. Head and Neck MCC Prognostic Factors

Differences in tumor site and characteristics have implications for metastasis, recurrence, and survival. Lip tumors, scalp tumors, local invasion, nodal metastasis, and distant metastasis are all associated with increased mortality [4,18]. Although nodal metastasis is a prognostic factor, single *versus* multiple positive lymph nodes did not affect survival. Patients without a regional lymph node dissection or elective radiation have a significantly decreased 5-year survival [4].

Unlike melanoma, tumor thickness is a poor predictor of clinical stage and metastatic potential. In a review of 34 patients with MCC of all body sites, there was no difference in tumor depth between stage 1 and stage 3 patients [19]. Although thickness is poorly correlated with regional metastasis, tumor thickness >10 mm has a decreased survival [20]. Primary tumor size and lymphovascular invasion have been linked with increased risk of regional metastasis, but only lymphovascular invasion has been correlated with decreased survival [4,19].

### 2.2. Outcomes

MCC has an overall 5-year survival of 30%–64% for all body sites, which varies significantly if there is regional or distant metastasis [21]. In a study of 37 patients treated with a combination of surgery and radiotherapy with or without chemotherapy, there was a 48% overall 5 year survival for stage I disease and 18% for stage II disease. Of this cohort, one of eight patients was successfully salvaged after recurrence [15]. A significant survival advantage was noted for patients who underwent regional lymph node dissection compared to those who had treatment of only the primary site (70% increased risk of death without nodal dissection) [4]. There is a 75%–78% local and regional lymphatics control rate after RT alone or combined with surgery [15,22,23]. When distant metastasis is present, there is an average 9 month survival [21].

## 3. Diagnosis and Work-up

Diagnosis and evaluation of MCC requires a thorough history and physical examination focusing on extent of the primary tumor, regional lymphatics, satellite metastases, and history of immunosuppression. Unusual appearing lesions should be biopsied due to the innocuous presentation of MCC. Imaging should include the primary tumor, regional lymphatics, and lungs. A PET-CT is often helpful in staging and treatment planning [15].

### 3.1. Staging

MCC is staged using the TNM classification via factors of tumor dimension, invasion, micro and macroscopic lymph node metastasis, satellite metastasis, and distant metastasis (Table 1). Stage I and II include primary tumors of <2 cm (T1) or >2 cm (T2, T3, & T4) respectively without metastasis (Table 2). Stage III disease indicates lymph node metastasis, and stage IV has distant metastasis [24]. Stages I and II are subdivided into A and B based on whether the regional lymph nodes are pathologically or clinically staged as N0. Stage IIC classifies T4 tumors with N0 lymph nodes. Stage IIIA has micrometastatic lymph node metastasis and stage IIIB has macrometastases to the regional lymph nodes [20].

**Table 1:** AJCC 2010 TNM staging System for Merkel Cell Carcinoma [24].

Primary Tumor	Regional Lymph Nodes	Distant Metastasis
Tx—Primary tumor cannot be assessed	Nx—lymph nodes cannot be assessed	Mx—metastasis cannot be assessed
T0—No evidence of primary tumor	cN0—Nodes negative by clinical exam	M0—No distant metastasis
Tis—*In situ* primary tumor	pN0—Nodes negative by pathologic exam	M1a—Metastasis to skin, subcutaneous tissues, or distant lymph nodes
T1—≤2 cm maximum tumor dimension	N1a—Micrometastasis: identified after LN biopsy	M1b—Metastasis to lung
T2—>2 cm but ≤5 cm maximum tumor dimension	N1b—Macrometastasis: clinically detectable nodes confirmed on biopsy	M1c—Metastasis to other visceral sites
T3—>5 cm maximum tumor dimension	N2—In-transit lesion: Tumor distal to primary lesion	
T4—Primary tumor invades bone, muscle, fascia, or cartilage		

**Table 2:** AJCC 2010 staging for Merkel Cell Carcinoma [24].

Stage		TNM Classification	
0	Tis	N0	M0
IA	T1	pN0	M0
IB	T1	cN0	M0
IIA	T2/T3	pN0	M0
IIB	T2/T3	cN0	M0
IIC	T4	N0	M0
IIIA	Any T	N1a	M0
IIIB	Any T	N1b/N2	M0
IV	Any T	Any N	M1

### 3.2. Utility of PET Scan

Fluorine-18-fluorodeoxyglucose positron emission tomography (PET) and PET-CT have been studied as a tool for staging of MCC with encouraging results. MCC is a fast-growing metabolically active tumor making it PET avid [25,26,27,28,29,30]. A review of 6 studies which included 92 patients with MCC of any site demonstrated a 90% sensitivity and 98% specificity for metastatic disease, except for a limitation in detection of brain metastasis due to the high background physiologic metabolic rate [25]. Another study of 16 patients comparing FDG-PET to ultrasound, CT, and MRI of various regions of the body showed improved specificity for PET (96.2% *vs.* 89.1%) but decreased sensitivity (85.7% *vs.* 95.5%) [31]. Compared to traditional PET, PET-CT has improved anatomic localization, with potential to improve accuracy [30]. Additionally, the results of pre-treatment PET scanning in 102 patients impacted management in 37%. Another retrospective study of 18 patients demonstrated altered staging in 33% of patients and changes in management in 43% due to PET-CT imaging [26,30,32,33]. Although PET is useful in detection of metastatic disease, it is unclear whether this improves survival [25,26,30].

Other nuclear medicine imaging used for neuroendocrine tumors, including F-DOPA PET and somatostatin receptor imaging [29], did not perform as well as F-FDG PET for MCC. The better performance of F-FDG PET is thought to be due to the high proliferation with increased glucose metabolism of MCC [25]. Somatostatin receptor scintigraphy has been associated with higher rates of false positive and negative results, and cannot be recommended for routine use.

### 3.3. Unknown Primary Tumor

A patient who presents with pathologic lymphadenopathy in the setting of an unknown cutaneous lesion is said to have an unknown primary tumor. It is unclear, but the affected lymph nodes may be from an undetected skin lesion, a *de novo* malignancy in the nodal basin, or from secondary involvement from a subdermal lesion [34]. Up to 12% of patients present with regional or distant metastasis with an unknown primary tumor [21]. Due to the few number of reported patients, there is a paucity of data in management recommendations for unknown primary tumors.

In 34 cases of unknown primary disease, 7/34 presented in the cervical lymph nodes [34]. In another study of 23 patients, four were found to have metastatic disease at presentation. When MCC is detected in a lymph node by FNA or excisional biopsy, work-up should begin with a staging PET-CT and a thorough skin exam for the involved draining lymph node basin. Treatment is similar to that of similarly staged known primary lesions. There have been conflicting reports on prognosis of patients with unknown primary tumors compared to known primary tumors, but there may be a survival advantage for those with unknown primary tumors (36.4% *vs.* 76.9%) [34]. Continued research is needed to better delineate treatment recommendations for these patients.

### 3.4. Role of Sentinel Lymph Node Biopsy

Treatment algorithms have international and institutional variation due to controversy in utility of sentinel lymph node biopsy (SNLB) when compared to elective radiation or neck dissection. Sentinel lymph node biopsy has gained acceptance for use in the head and neck for melanoma primary tumors, however its role in MCC is still under debate [35]. MCC is an aggressive malignancy that has high rates of local, distant, and in-transit lymph node metastasis, making its behavior similar to that of melanoma cutaneous malignancies. As discussed previously, in the clinically and radiologically N0 neck there is a risk of undetected micrometastasis to the draining lymph nodes. It is thought that identifying metastasis to the first “sentinel” lymph node would predict the presence or absence of malignancy in the entire lymph node basin. When the sentinel lymph node is positive with micrometastasis, there is a 25% chance of other lymph nodes harboring metastatic disease [21]. If the sentinel lymph node is negative, the remaining nodal basin tends to be monitored, while a complete neck dissection or radiation therapy is performed for a positive sentinel lymph node [36].

A clinical N0 neck is a poor predictor of negative metastasis, as 23%–32% of N0 necks have occult metastatic disease [21,37]. In melanoma, patients are selected for sentinel lymph node biopsy based on tumor depth and histologic characteristics such as ulceration and mitotic figures, however MCC does not share these histologic prognostic factors. Factors such as lymphovascular invasion, tumor size >2 cm, tumor thickness, and mitotic rate have been suggested, but even with negative risk factors, a high rate of neck metastasis exists [36]. Messina *et al.* studied 12 patients with MCC of all sites who underwent sentinel lymph node biopsy, of which 10 patients had a negative sentinel lymph node without nodal recurrence at 10.5 months [38]. This study, and studies with similar results [39,40] suggest the utility of sentinel lymph node biopsy in reducing unnecessary neck dissections and adjuvant therapy.

Other authors argue that due to the high rate of regional lymph node metastasis, a protocol of elective irradiation of all N0 regional nodes is advocated without lymph node biopsy [15,41,42]. In addition, other studies report high rates of regional lymph node failure after negative sentinel lymph node dissection. The head and neck has a unique lymphatic drainage system where there may be multiple sentinel lymph nodes due to multiple draining lymph node basins. Due to a paucity of data specifically relating to SNLB of the head and neck, more studies are needed to clarify its role.

## 4. Treatment

MCC is an aggressive tumor with best cure rates achieved with multimodal therapy. Wide local excision with regional lymph node dissection and adjuvant radiotherapy is advocated, with definitive radiotherapy reserved for unresectable tumors [36].

### 4.1. Primary Tumor

Wide local excision with generous margins are a cornerstone of reducing local recurrence rates [36,43]. In a review of 251 patients from a single institution, negative margins were obtained in 94% of patients with an average surgical margin of 1.1 cm [21]. Margins >1 cm do not appear to decrease local recurrence rates for small tumors. A 1 cm margin is recommended for tumors <2 cm and a 2 cm margin for tumors >2 cm [36,44]. Depth of incision should be to the investing fascia or pericranium and include frozen section margins [44]. The 12.5% successful salvage rate highlights the importance of aggressive initial locoregional management [15]. Immediate reconstruction of the surgical defect is preferred if it can be accomplished with a simple closure, but should be delayed after final histologic margins are assessed for complex reconstructions.

Radiation therapy alone is indicated for unresectable disease, but combination therapy with excision and adjuvant radiation likely results in improved local-regional control [15,22,23,45,46]. In a study comparing 44 patients who underwent monotherapy (either surgery or RT) and 66 patients who had combination surgery with adjuvant RT, there was a statistically significant benefit in local control, regional control, and disease-free survival in the combined therapy group after 2.3 year mean follow-up [46].

### 4.2. Treatment of Lymph Nodes

There is a clear need to address occult metastasis, but there is no consensus about the best algorithm. Options include electively irradiating all N0 necks, surgical neck dissection, or using SLN biopsy to guide the need for therapy. A randomized controlled trial of 83 patients with stage I MCC of all body sites was performed where patients were randomized to elective radiation of nodal basins *versus* observation of these nodal basins. The treatment arm in this study showed a significant improvement in probability of regional recurrence in the treatment arm (0% *vs.* 16.7%), however failed to show a survival benefit [47]. Twenty-six patients who underwent SLNB with microscopically positive disease had 100% 2 year control rate whether treated with RT alone (19 patients) or with neck dissection with or without adjuvant RT (7 patients) [22]. This suggests that the clinically N0 neck can be effectively managed with monotherapy. Failure to address the nodal basins in a clinically N0 neck resulted in a 33% failure rate, which is significantly higher than those who were treated with elective nodal RT [48].

Macroscopically positive lymph nodes had a 73%–78% control rate whether treated with RT alone or with combination therapy, and are best treated with either RT or neck dissection. A better outcome is reported for occult metastases that receive >50 Gy and >55 Gy for gross disease [48]. Distant metastatic disease is out of the scope of discussion of this review, however patients should be carefully evaluated in a multidisciplinary tumor board with consideration given to a combination of surgery, radiotherapy, or chemotherapy [44].

### 4.3. Role of Chemotherapy

Trials of chemotherapy (carboplatin and etoposide) have been done in a non-controlled study which included 40 patients with high-risk features of recurrent disease, positive lymph nodes, tumor >1 cm, and positive surgical margins. In this trial, patients received concurrent chemoradiation with no benefit seen for survival, local-regional control, and distant metastasis free survival when compared to a historical control treated with surgery and radiation alone [41]. This data suggests that the addition of chemotherapy may not be beneficial in a patient without known metastatic disease.

## 5. Discussion

Merkel cell carcinoma is an aggressive neuroendocrine cutaneous malignancy with a predilection for regional and distant metastasis. This malignancy presents most commonly on the head and neck of elderly Caucasian males with a higher prevalence in the immunosuppressed. A high index of suspicion must be maintained due to the often asymptomatic presentation. Lip tumors, scalp tumors, local invasion, nodal metastasis, distant metastasis, and lymphovascular invasion are poor prognostic factors.

Due to a worse prognosis for recurrent tumors, the best chance to achieve locoregional control is with appropriate initial treatment. Studies have shown a benefit to combination surgery with adjuvant radiation as initial treatment for resectable tumors. Surgery should include wide 1 cm margins of the primary tumor. Radiotherapy [15] alone is used for unresectable disease as chemotherapy has no proven benefit in regional or distant control.

The prevalence for metastatic disease makes accurate staging important for management. PET-CT has proven to be both sensitive and specific, and is justified for use in initial staging and surveillance [25].

The regional lymphatics must be addressed due to the high occult metastasis rate and poor survival of patients managed with treatment of the primary tumor alone. There is no consensus about the best treatment algorithm for the N0 regional lymphatics, but data suggests that elective neck dissection, elective radiotherapy, or a sentinel lymph node biopsy to guide management of the nodal basins is effective in improving regional control.

## 6. Conclusions

Due to the rarity of MCC, it is difficult to make definitive management recommendations, however it is clear that an early aggressive management of the primary tumor with combination surgery and adjuvant RT affords the best outcomes. The tumor is likely to metastasize, and PET-CT is sensitive in detection of metastatic disease. There is a high incidence of occult metastatic disease, so even in the clinically N0 necks, the regional lymph nodes must be addressed with either elective radiation, elective neck dissection, or sentinel lymph node biopsy to guide management. Chemotherapy has no proven benefit in locoregional control and is reserved for metastatic disease.
